# Characterising the biophysical properties of normal and hyperkeratotic foot skin

**DOI:** 10.1186/s13047-015-0092-7

**Published:** 2015-08-12

**Authors:** Farina Hashmi, Christopher Nester, Ciaran Wright, Veronica Newton, Sharon Lam

**Affiliations:** School of Health Sciences, Centre for Health Sciences Research, University of Salford, Manchester, UK; Foot and Ankle Research Programme, Centre for Health Sciences Research, School of Health Sciences, University of Salford, Manchester, UK; School of Health Sciences, University of Salford, Manchester, UK; Reckitt Benckiser, Dansom Lane, Hull, UK

**Keywords:** Callus, Corns, Xerosis, Heel fissures, Dry skin, Plantar foot skin hyperkeratosis, Biophysical parameters, Skin classification (SELs), Quantification

## Abstract

**Background:**

Plantar foot skin exhibits unique biophysical properties that are distinct from skin on other areas of the body. This paper characterises, using non-invasive methods, the biophysical properties of foot skin in healthy and pathological states including xerosis, heel fissures, calluses and corns.

**Methods:**

Ninety three people participated. Skin hydration, elasticity, collagen and elastin fibre organisation and surface texture was measured from plantar calluses, corns, fissured heel skin and xerotic heel skin. Previously published criteria were applied to classify the severity of each skin lesion and differences in the biophysical properties compared between each classification.

**Results:**

Calluses, corns, xerotic heel skin and heel fissures had significantly lower levels of hydration; less elasticity and greater surface texture than unaffected skin sites (*p* < 0.01). Some evidence was found for a positive correlation between hydration and elasticity data (*r* ≤ 0.65) at hyperkeratotic sites. Significant differences in skin properties (with the exception of texture) were noted between different classifications of skin lesion.

**Conclusions:**

This study provides benchmark data for healthy and different severities of pathological foot skin. These data have applications ranging from monitoring the quality of foot skin, to measuring the efficacy of therapeutic interventions.

## Background

Plantar skin has a unique structure compared to skin on other parts of the body [1]. Its role is to withstand and adapt to the external stresses during physical activities. As a result the epidermis, in particular the stratum corneum (SC), has evolved structural specialisations (such as specific spatially arranged keratins) to provide resistance to physical stresses [1]. Hypertrophy of the SC, known as hyperkeratosis, is one of the primary protective responses and thought to be triggered by increases in external mechanical stresses. During hyperkeratosis dermal and epidermal cells react to stress by generating inflammatory cytokines which cause the incomplete differentiation of corneocytes. This accelerates transit through the epidermis, rendering the SC biochemically and structurally compromised [2]. Examples include the incomplete degradation of desmosomes and abnormal lipid layer formation, which lead to altered corneocyte adhesion and desquamation, and SC thickening [3, 4].

In the foot hyperkeratosis presents as calluses, corns and heel fissures [5, 6]. Up to 78 % of people have corns and callus [5] with even higher rates in older people [6–10]. Hyperkeratosis in cases of diabetes is strongly associated with increased risk of foot ulceration [11–13] and in older people it impacts on balance [14, 15]. Whilst multifactorial in origin, without data describing the biophysical characteristics of these skin lesions, it is impossible to explain how internal and external factors lead to their formation. Nor is it possible to explain how their structure and function impacts upon foot health and thereafter mobility.

A logical starting point is whether factors that influence the function of plantar skin, such as hydration, elasticity and surface texture, are significantly different in hyperkeratotic lesions. The SC is not a ‘dead layer’ as it consists of inter and intra cellular components, such as Natural Moisturising Factors (NMF) and lipids, that provide protective and adaptive functions [16]. These components and the arrangement of corneocytes in the SC sustain water content which is vital for enzyme activity; plasticity; resistance to shear; desquamation and skin pH. In short, water is integral in maintaining SC homeostasis. From a clinical point of view appropriately hydrated SC appears pliable and devoid of scaling or dryness. When desiccation of the SC occurs, the degradation of corneodesmosomes is impaired leading to ‘clumping’ of the corneocytes, producing the appearance of roughness, flaking and scaling. This process could explain what is taking place on the periphery of heel skin and fissures, but in the case of plantar corns and calluses weight bearing forces are additional contributory factors. It should be noted that although the SC is the first point of contact of external stresses, the dermal and subdermal tissues play a role in dissipating these stresses. Therefore, an understanding of how the plantar soft tissues respond to external forces (i.e. its elasticity) is relevant in addition to evaluating skin surface (SC) hydration. Furthermore, it seems logical that a relationship might exist between hydration and skin texture in foot skin too. However, these features of normal, callused and fissured plantar skin have not yet been reported nor the expected interrelationships explored.

Despite the lack of objective data characterising corns, callus and fissures, sufferers are often advised to use keratolytic and humectant based emollients, to alter the hydration and desquamation rates of SC cells [17–22]. Although we have some idea of how effective these treatments are, the efficacy and dose recommendations have not been systematically explored. Furthermore, the extent to which treatments return skin to normal is unknown. Finally, optimisation of existing treatments and systematic development of new intervention strategies is impossible without a more thorough characterisation of the underlying hyperkeratotic lesions.

Criteria have been proposed to stratify the severity of callus and fissures [23, 24]. In the absence of objective data on these lesions this seems premature, but this could assist in monitoring skin lesion progress. However, these are based entirely on subjective clinical observation rather than objective data describing the properties of the skin. The validity of classification approaches is therefore untested. No classification has been suggested for heel fissures, though related work on xerosis elsewhere on the body (e.g. arms and legs) could be transferrable. Again, quantitative data on the properties of the heel fissures could enhance any clinical classification.

This study aimed to quantitatively characterise the biophysical properties of normal plantar skin and hyperkeratotic plantar lesions. We aimed firstly to measure four different physiological and functional parameters of foot skin (skin surface (SC) hydration, epidermal and subdermal elasticity, dermal collagen and elastin fibre organisation, and skin surface texture) on normal foot skin, corns, calluses, heel fissures, and xerotic heel skin. A second aim was to test existing schemes for skin lesion classification, by comparing the biophysical properties of the different severities of each skin lesion.

## Methods

The study protocol was reviewed and approved by the institutional Ethics Committee at the University of Salford (application number HSCR12/55). People with calluses, corns, heel fissures or xerotic heel skin were purposefully recruited via a local newspaper advertisement. All participants provided written informed consent prior to taking part. The study was conducted at one academic clinical site (University of Salford).

### Inclusion criteria

Participants were eligible if they were aged 18 years or over and reported a history of plantar corns, calluses, heel fissures and xerotic skin that had not been treated in the previous six weeks. The selection of the skin conditions was achieved using clinical assessment criteria currently used in practice [23, 24].

### Exclusion criteria

Participants were excluded if they presented with skin disorders affecting the foot such as infections (e.g. Athlete’s foot), dermatitis, psoriasis, un-healed skin wounds, ulcers or blisters. Open heel cracks (i.e. with exposed dermis) were also excluded. Any participant with a known systematic disease including peripheral vascular disease or musculoskeletal disorders of the foot or ankle, rheumatoid arthritis or diabetes was excluded.

### Biophysical measurements

Prior to performing measurements participants acclimatised to room temperature and humidity conditions for at least 15 min. The participant sat on a plinth with legs extended and the plantar aspects of both feet facing the investigator. The environmental conditions were not directly controlled; however the room was selected due to the minimal variation in temperature and humidity throughout the day and from day-to-day. Relative humidity (RH) and room temperature were monitored and recorded during the test procedures, via sensors incorporated in each of the devices. Temperature and RH values were recorded each time a measurement was taken using the probes. The average values were calculated for each day and compared using the Mann Whitney U test for day-to-day differences in environmental conditions and differences between the morning and afternoon data collection sessions.

The SC hydration was measured using a Corneometer® CM (arbitrary units, AU). Ten repeated measures were taken at each skin site and an average value calculated. Collagen and elastin fibre organisation (CEFO) was measured using a Reviscometer® RVM 600 (arbitrary units, AU). Twenty repeated measures were taken and a mean value calculated. This measures the anisotropy of collagen and elastin fibres in the dermis, providing a surrogate measure of viscoelasticity. Measurement of CEFO on the periphery of the heel was not conducted due to the curved architecture of the foot in this region. Only tissue with a relatively flat surface contour can be evaluated reliably using this device. Skin elasticity in response to suction pressure was quantified using the Cutometer® 580 MPA. The maximum displacement of the skin after 30 s of 500 mbar negative pressure application was recorded. Although the ideal way to represent the data would be to calculate the *stiffness* of the skin, this was not possible as the thickness of the skin before and after the application of force was not measured. Therefore, the next appropriate option was to calculate the *elasticity* of the skin using the following equation:

$$ \mathrm{Elasticity} = \frac{\mathrm{deformation}\ \mathrm{of}\ \mathrm{the}\ \mathrm{tissue}\ \left[\mathrm{mm}\right]}{\mathrm{force}\ \mathrm{applied}\ \left[\mathrm{mbar}\right]} $$

The elasticity data was reported in mm/mbar [25].

Skin surface texture was imaged and analysed using the contrast parameter of the Visioscan® VC 98. This measures surface homogeneity with greater values reflecting greater contrast between pixels and therefore ‘rougher’ skin. A detailed description of the devices, their reliability and sensitivity for foot skin, and measurement procedures have been reported [26]. All devices were obtained from Courage-Khazaka Electronic GmbH (Cologne, Germany).

**Fig. 1:**
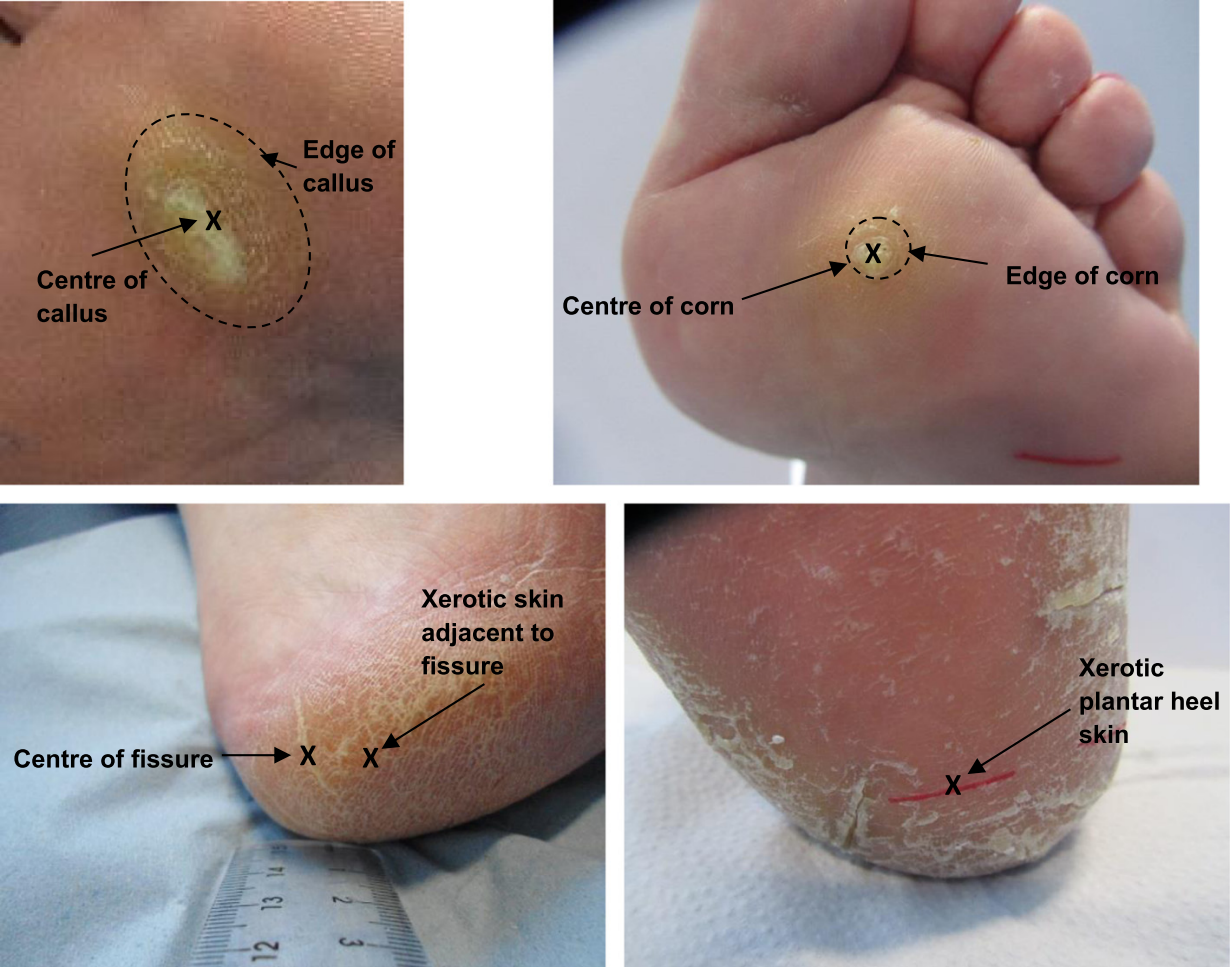
Measurement landmarks used for plantar calluses, plantar corns, heel fissures, and xerotic plantar heel skin

**Fig. 2:**
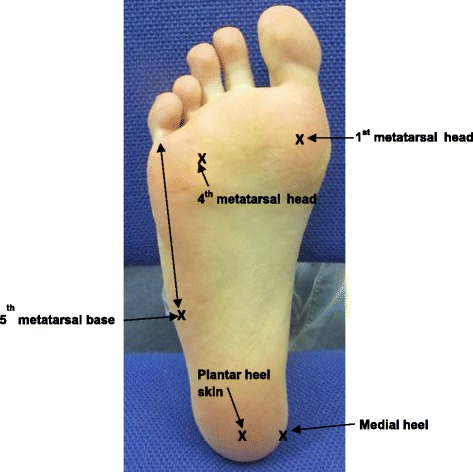
Measurement landmarks used for normal skin sites. The 5th met. base was located by palpation and the skin site marked by measuring 1 cm inward from the lateral boarder of the foot. The 1st or 4th PMA was also located by palpation and marking the region at the centre of the joint between the proximal phalanx and the metatarsal. The plantar aspect of the heel was marked 2 cm inwards from the posterior centre of the heel. The medial heel skin site was in alignment with the plantar heel mark

Skin parameters were measured at specific sites (Fig. 1): centre and edge of callus and corn; centre of heel fissure; and xerotic skin adjacent to the fissure and xerotic plantar heel skin. Measures were also taken from normal (i.e. unaffected) skin adjacent to the skin lesions at least 2 cm away from the edge of the callus (Fig. 2): 1st or 4th plantar metatarsal area depending on the site of the callus/corn (PMA), and base of the 5th metatarsal (5th met. base). Measurements were taken from one or both feet. For corns one texture image and value was derived since the entire lesion always fitted into one Visioscan® image.

### Digital photographs of skin lesions

Images of the skin lesions were captured using a digital camera (Sony Cyber – shot DSC – W510). A standard protocol was used for every photograph captured. A white card was placed behind the foot before capturing the image. Each photograph was taken at a distance of 10 cm away from the foot. All attempts were made to ensure that the front of the camera was perpendicular to the bottom of the foot. The flash and zoom facilities were not used, i.e. factory settings were used. The same lighting in the research room was used for every data collection session.

### Classification of heel fissure and plantar callus

#### Classification using existing clinical criteria

Subjective assessments were made using published grading systems [23, 24] by an independent assessor (podiatrist with 22 years clinical experience and not involved with data collection). Photographs of the calluses and corns were rated by the assessor using the existing clinical criteria [23]. For callus, photographs were rated by an independent researcher according to criteria from Merriman [23]: grade 1 “no specific callus plaque, but diffuse or pinch callus tissue present or in narrow bands”; and grade 2 “circumscribed, punctate oval or circular, well-defined thickening of keratinised tissue”.

There is no clinically validated classification system for heel fissures; therefore the nine point (0–8) Xerosis Assessment Score (XAS) was used [24]. This is commonly used to assess the amount and size of skin flakes, scales and fissures on the body, including the legs [24] but not the foot. The scoring scale is: 0: normal skin; 1: few minute flakes; 2: many places many undifferentiated flakes; 3: some polygonal scales; 4: moderate number of polygonal scales; 5: large number of polygonal scales; 6: fissuring between scales; 7: moderate fissuring between scales; and 8: deep fissuring between scales.

#### Sub-classification of the existing clinical criteria

In order to determine whether the biophysical outcome measures where sensitive enough to detect differences within classification grades and therefore potentially enhance the differentiation of callus severity the following sub classifications were formulated. Grade 2 calluses [23] were further subdivided into ‘moderate’ and ‘heavy’ classifications based on the relative thickness of the callus plaque. Grade 1 [23] callus was therefore considered ‘light’ callus on the new 3 point scale.

The XAS [24] was adapted to describe a more realistic clinical view of xerotic and fissured heel skin. Three grades of xerotic heel skin severity were formulated: grade 1 = XAS 1–4 (xerotic skin with indistinct fissures); grade 2 = XAS 5–6 (shallow fissured skin); grade 3 = XAS 7–8 (moderate to deep fissured skin).

Photographs of the calluses and fissures were rated according to these criteria by second independent assessor (podiatrist with 22 years clinical experience and not involved with data collection).

### Statistical analysis

The objective biophysical outcome data (for hydration, elasticity and texture only) was grouped to correspond with the different grades of calluses and fissures according to the current grading systems [23, 24] and the sub classification of these criteria described in the previous section. Appropriate statistical tests were conducted to assess similarities and differences in outcome measures for the different grades of hyperkeratosis, described as follows. Normality assumptions were tested using the Kolmogorov - Smirnov test, which indicated that the distribution of the data departed significantly from normality (*p* < 0.05) therefore, non-parametric tests were used. Descriptive data were expressed as medians and interquartile ranges. Wilcoxon and Mann Whitney tests were used for pairwise comparisons and Spearman’s correlation coefficient was used for correlation analyses. Statistical analyses were carried out in SPSS 20. All statistical tests were performed using a two-tailed 5 % overall significance level.

## Results

Ninety three volunteers with a mean age of 47.8 years (range: 20 – 78 years) were recruited (Table 1). The majority were females (81 %). All participants presented with one or more of the hyperkeratotic conditions and one or both feet were included for measurements depending on the presentation of the conditions. Therefore, of the 93 participants 34 people had heel fissures (61 feet with fissures only and 7 feet with fissures and corns were measured); 46 people had calluses (61 feet measured, i.e. 15 people had both feet measured and 31 people had one foot only measured) and 13 people had corns only (13 feet were measured). All hyperkeratosis groups were balanced except for age where the corn group was significantly older than callus and fissure groups (*p* = 0.05 and *p* = 0.01, respectively).

**Table 1:** Baseline characteristics of all participants

	Skin sites from hyperkeratosis skin group (*n* = 93)^a^				
Variable	Callus (*n* = 61)^b^	Corn (*n* = 20)^b^	Heel fissures (*n* = 68)^b^	Xerotic skin adjacent to fissure (*n* = 68)^b^	Xerotic plantar heel skin (*n* = 68)^b^
Age (yr)					
Median	48	54	45.5	45.5	45.5
Min, max	23, 78	28, 74	20, 74	20, 74	20, 74
IQR	24	27	23	23	23
Sex (% female)	84	80	81	81	81
Height (m)					
Median	1.64	1.65	1.68	1.68	1.68
min, max	1.32, 1.98	1.42, 1.80	1.52, 1.89	1.52, 1.89	1.52, 1.89
IQR	0.19	0.10	0.17	0.17	0.17
Weight (kg)					
Median	73.70	67.10	76.2	76.2	76.2
Min, max	46.30, 111.60	53.00, 83.00	55.0, 123.0	55.0, 123.0	55.0, 123.0
IQR	33.47	20	17.0	17.0	17.0
BMI (kg/m^2^)					
Median	28	25	28	28	28
Min, max	19, 41	19, 30	19, 46	19, 46	19, 46
IQR	7	8	6	6	6

^a^Represents number of people and ^b^refers to the number of feet

The environmental variables at the time of the study were: 23.6 ± 1.0 °C for the room temperature and 53.1 ± 8.4 % for the RH. There were no statistically significant differences in these variables from day to day (*p* > 0.42) or between morning and afternoon data collection sessions.

### Biophysical properties of skin

**Fig. 3:**
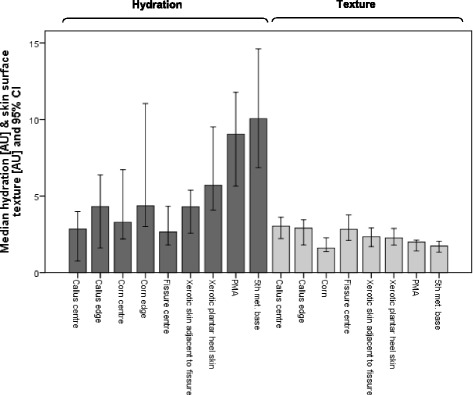
Hydration (AU) and skin surface texture (AU) for all skin sites. Data is expressed as median and 95 % CI. Hydration: the greater the value the more hydrated the SC; skin surface texture: the lower the value the smoother the skin

**Fig. 4:**
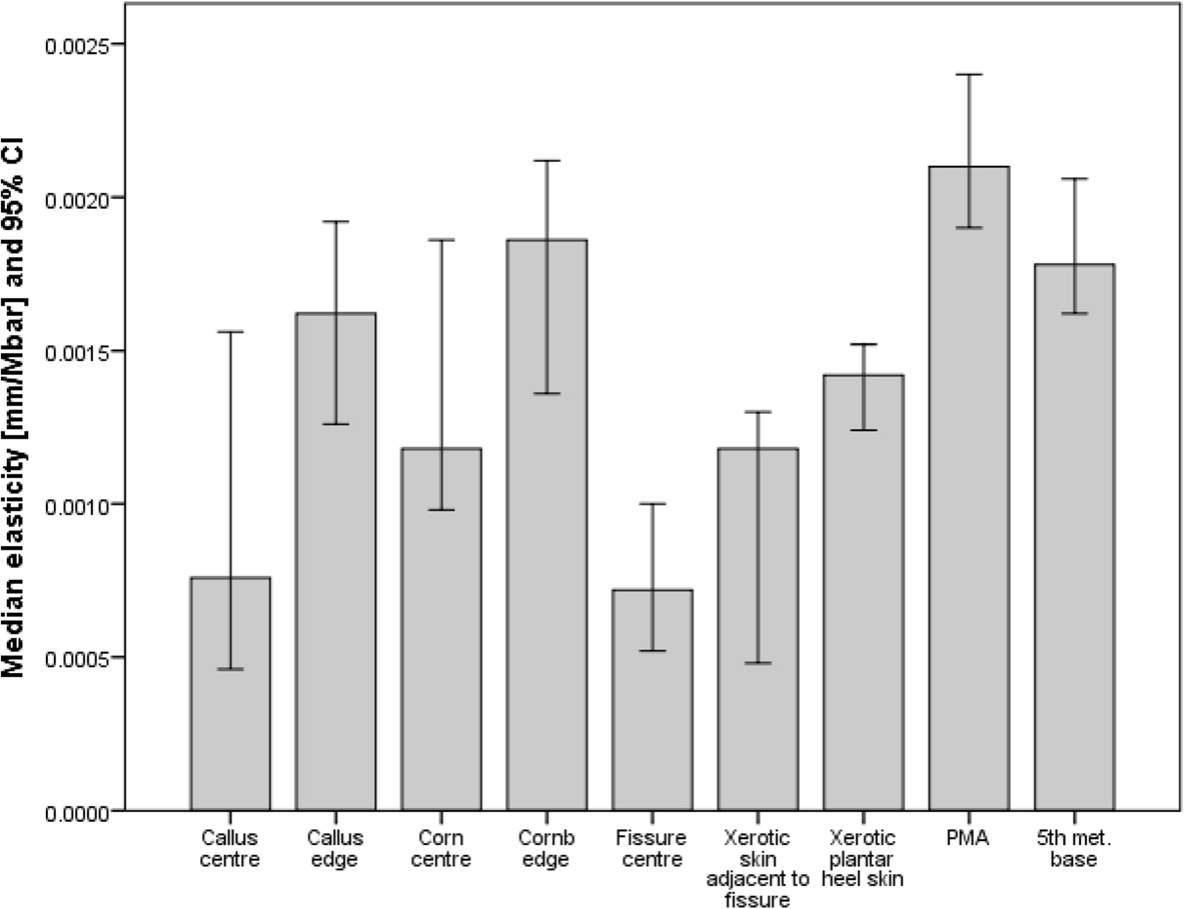
Elasticity of all skin sites (mm/mbar). Data is expressed as medians and 95 % CI. The higher the value the more elastic the skin

**Fig. 5:**
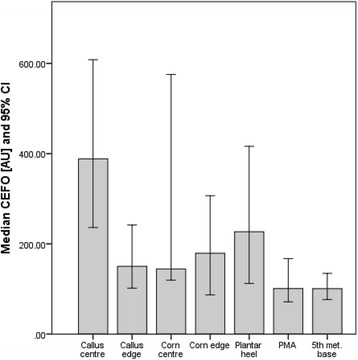
Collagen and elastin fibre organisation of all skin sites (AU). Data is expressed as medians and 95 % CI. The higher the value the less organised the collagen and elastic fibres and the less elastic the skin

Figures 3, 4 and 5 illustrate the biophysical measures from all skin sites. All lesions showed significant differences (*p* ≤ 0.01) from normal skin sites and each other except: centre versus edge of corn tissue (CEFO); callus centre versus corn centre (CEFO and hydration) and normal PMA versus normal 5th met. base sites (hydration).

#### SC hydration

The normal sites (PMA and 5th met. base) were approximately 4 times more hydrated than the centre of heel fissures and 3 times more hydrated than the centre of corns and calluses.

#### Elasticity

The normal sites (normal PMA and 5th met. base) exhibiting greater (approximately 2 times greater) elasticity than all hyperkeratotic sites. The least elastic site was the centre of the heel fissure where the elasticity was 50 % less than the adjacent xerotic skin. The centre of the corn tissue was significantly more elastic than the centre of callus tissue (median [IQR]: 7.6 × 10^−4^ [1.3 × 10^−3^] mm/mbar and 11.8 × 10^−4^ [0.94 × 10^−3^] mm/mbar, respectively).

#### CEFO

The normal sites (PMA and 5th met. base) demonstrated the lowest readings (approximately 50 % less than other sites). The centre of callus and corn tissue generated the greatest readings with the values being 4 times greater for callus tissue and 1.5 times greater for corn tissue.

#### Skin surface texture (contrast parameter)

Calluses and fissures had the greatest contrast (i.e. greatest roughness in texture), approximately 50 % greater than those from corn, PMA and 5th met. base sites.

### Correlations between biophysical properties

Correlation between hydration and elasticity was statistically significantly in 6 of the 9 tests (Table 2). The strongest correlation was 0.65 at both the centre and skin adjacent to heel fissures. Correlation between hydration and elasticity was also moderate at the centre of the callus (*r* = 0.56) but not at the callus edge (*r* = 0.29), nor normal skin. One of the 9 tests between hydration and collagen organisation was statistically significant (edge of corn, *r* = 0.54).

**Table 2:** Correlation analysis results

		Correlation: *r* value (significance level^a^)							
Pairwise correlation variables	Callus centre	Callus edge	Corn centre	Corn edge	Fissure centre	Xerotic skin adjacent to fissure	Xerotic plantar heel	PMA	5th met. base
Hydration v elasticity	0.56 (0.01)	0.29 (0.05)	−0.09	−0.19	0.65 (0.01)	0.65 (0.01)	0.34 (0.01)	0.13	0.25 (0.01)
Hydration v collagen organisation	0.11	−0.21	−0.25	0.54 (0.05)			−0.17	0.14	−0.11
Collagen organisation v elasticity	−0.01	0.13	0.33	−0.45			−0.02	0.03	0.00
Skin surface texture and hydration	−0.18	0.00	−0.30		0.15	0.08	0.19	0.09	0.00

^a^Significance values are quoted where appropriate

### Classification of callus and fissures

**Fig. 6:**
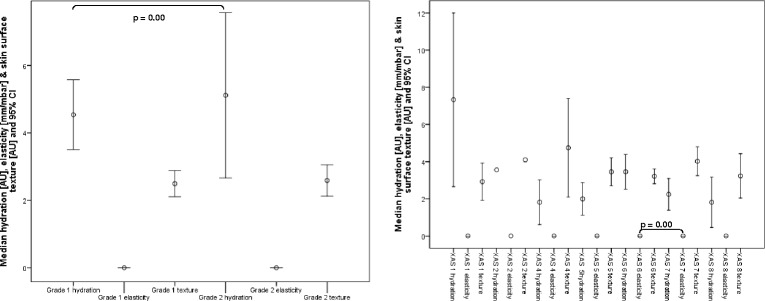
Graphs reporting the hydration, elasticity and skin surface texture values for Grade 1 and 2 callus according to Merriman (first graph) and Grade 1 to 9 heel fissures (second graph). P values of less than 0.01 are reported only

**Fig. 7:**
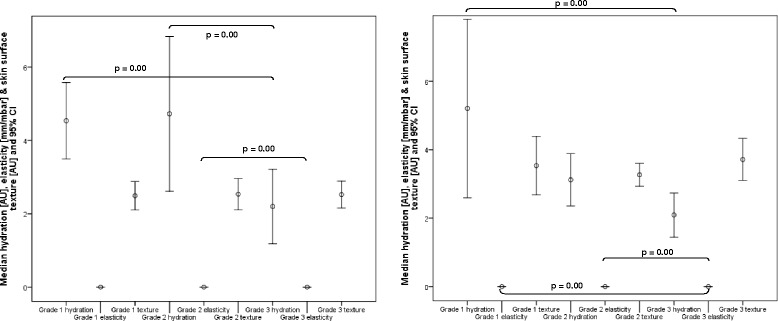
Graphs reporting the hydration, elasticity and skin surface texture values for new Grade 1 to 3 callus (first graph) and new Grade 1 to 3 heel fissures (second graph). P values of less than 0.01 are reported only

Figures 6 and 7 report the biophysical data for the current and adapted classification for calluses and heel fissures. According to the current callus classification system there were no significant differences in elasticity and texture between grades 1 and 2, but there was for hydration (*p* = 0.00). In the case of the adapted grading system, there was no significant difference between grade 1 (light) and grade 2 (moderate) callus groups in terms of hydration, elasticity nor texture. Texture was also not significantly different between moderate and heavy (grade 3) callus groups, but hydration and elasticity measures were significantly different.

For heel fissures, there were no significant differences between all the XAS grades in terms of hydration, elasticity and texture except for elasticity measures between grades 6 and 7 (*p* = 0.00). For the adapted grading system there was no significant difference between grade 1 (xerotic skin with indistinct fissures) and grade 2 (shallow fissures) in terms of hydration, elasticity nor texture. Texture was also not significantly different between grades 2 (shallow fissures) and 3 (moderate to deep fissures), and 1 (xerotic skin with indistinct fissures) and 3 (moderate to deep fissures).

## Discussion

The results show that measurable differences exist between normal and hyperkeratotic foot skin and that measures of hydration and elasticity were different between different classifications of callus and fissures.

### Biophysical characteristics of hyperkeratotic foot skin lesions

The centre of callus tissue is approximately 73 % less hydrated as corresponding normal plantar skin. This is not a surprising result as it is acknowledged that the biochemical and physical processes involved with hyperkeratosis lead to tissue desiccation as well as impaired differentiation and desquamation [1]. The hydration does not appear to be uniform across the surface the callus, as the data shows a significant increase in hydration (6 %) at the callus edge compared to the centre. The elasticity readings also show similar trends, which would support the notion that callus on the periphery of the plaque is relatively thinner (and therefore more flexible) than the central callus. One advantage that imaging would have over this method is the ability to confirm how much of the callus is protruding above or depressing below the level of the epidermis, providing a better understanding of the proportions of the epidermal, dermal and subdermal tissues that are being evaluated for elasticity. Information of this kind would add to the current body of knowledge identifying callus as a relatively inelastic ‘foreign body’ causing pressure wounds. The Cutometer® used in our study is unable to differentiate between elasticity of the epidermis and the dermis. Calluses demonstrated the greatest degree of skin surface roughness compared to fissures and corns.

In the case of plantar corns the hydration values were similar to those taken from the centre of callus plaques. However, elasticity data from the centre and edge of the corns and the levels of hydration on the corn edges were significantly greater compared to corresponding callus sites. This suggests that the corn tissue is more flexible than callus tissue; however through clinical experience it is clear that plantar corns have nuclei that advance deep into the epidermal architecture. It is also clear, via clinical observation, that the border between the edge of a corn and normal skin is often more distinct that that of a callus plaque. The corn is therefore embedded within a relatively soft and pliable normal plantar tissue. With this in mind the placement of the measurement probes on the edge of the lesions becomes challenging. If half the area of skin under the probe is corn tissue and the other half adjacent healthy tissue, then it is likely that the latter would skew the data.

The centre of heel fissures are the most desiccated and least elastic of all the lesions evaluated in this study. In contrast the adjacent xerotic skin sites, although exhibiting similar textural appearances to those of calluses and fissures, were more hydrated and more elastic than callus tissue.

### Correlations between biophysical properties

As would be expected there is a positive correlation between hydration and elasticity of plantar skin and hyperkeratotic lesions, however there is no correlation between skin hydration and surface texture. Interestingly, the normal PMA does not demonstrate a correlation between hydration and elasticity. The hydration and elasticity measures from PMA skin showed a greater variability than those from the hyperkeratotic lesions. This would suggest that the lesions are structurally and functionally less variable from person to person than normal plantar skin.

### Classification of callus and fissures

In the case of callus, the biophysical data suggests that our ‘light’ and ‘moderate’ callus classification should be merged to give two overall callus grades. The former is equivalent to grade 1 described by Merriman [23]. However our data has shown distinct differences in the nature of calluses within grade 2 of Merriman’s scale, i.e. differences between moderate and heavy callus, justifying the separate classification of these lesions.

The biophysical data only partially support the fissure classifications based on the subjective criteria of the XAS [24]. When the data was grouped to correlate with the progressive deterioration of heel skin (dry skin with indistinct fissures (grade 1) to shallow fissures (grade 2) to moderate/deep fissures (grade 3)), there were statistically significant differences between grades 2 and 3 for the hydration and elasticity measures. As with the callus classification, our grades 1 and 2 can be merged, therefore providing a 2 grade system.

Although texture has been shown to be valuable in detecting differences between different hyperkeratotic lesions the results in the context of classification reveal it to be a poor differentiator of both callus and fissure severity and should therefore be excluded from the classification process.

### Limitations

The capacitance method of measuring skin surface hydration quantifies water content to a depth of 0.45 mm [27]. According to Magnetic Resonance Imagining data of heel skin [28] the SC thickness is approximately 0.28 mm. The absence of thickness data for the other plantar skin sites, our data reflects hydration of superficial SC only for calluses, corns and fissures and not the full thickness of the lesions themselves.

We used acoustic sound wave propagation to measure skin elasticity. Although this was shown to be reliable in our previous work [26], the standard error of measurement was relatively high compared with other techniques. The variability between people in the callus and corn groups was particularly high and some readings from these sites were above the recommended limits of measurement of the device [26]. Although other researchers have found a strong correlation between Cutometer® and Reviscometer® measurements [29] our data did not show a correlation between the two variables.

The aim of this study was focused on understanding the structural and functional nature of hyperkeratotic foot skin in more detail, therefore the design was directed at including a range of severities of lesions according to the existing criteria. Pain levels were not recorded for this work and it would seem appropriate to test this in the future.

## Conclusions

This study has shown that distinct biophysical differences exist between normal and hyperkeratotic foot skin lesions. Some sub classification of callus and fissures is possible using hydration and elasticity but not texture data.
